# Prevalence of metabolic hyperferritinemia and association between ferritin and insulin resistance in persons with impaired glucose regulation in the HUNT2 population study

**DOI:** 10.1186/s13098-026-02161-9

**Published:** 2026-04-25

**Authors:** Kristoffer Sand, Robert Brudevold, Torstein Hole

**Affiliations:** 1 Department of Medicine, Ålesund Hospital Møre og Romsdal Hospital Trust, Ålesund, N-6026 Norway; 2 Department of Health Sciences Ålesund, Norwegian University of Science and Technology (NTNU), PB 1517, Ålesund, N-6025 Norway; 3 Faculty of Medicine and Health Sciences, Norwegian University of Science and Technology (NTNU), Trondheim, N-7491 Norway

**Keywords:** Ferritin, Insulin resistance, Hyperferritinemia, Metabolic syndrome, Metabolic hyperferritinemia

## Abstract

**Background:**

Several reports have indicated an association between insulin resistance and hyperferritinemia, and a recent consensus statement has suggested the term metabolic hyperferritinemia (MHF) for patients with metabolic syndrome and increased serum ferritin. The objective of this study was to examine the association between serum ferritin levels and insulin resistance in an unselected Norwegian population.

**Methods:**

Two thousand people were randomly selected from the public registry in one municipality in Nord-Trøndelag County, Norway. Of the respondents, 1115 completed a screening glucose tolerance test to determine the prevalence of impaired glucose tolerance (IGT)/type 2 diabetes mellitus (T2DM), and these individuals were then selected for further examination. A control group comprising 100 age- and gender-matched individuals with normal glucose tolerance and CRP less than 5 was retrospectively generated from the same cohort.

**Results:**

One hundred and seventy-six people were diagnosed with impaired glucose tolerance or diabetes mellitus type 2 (IGT/T2DM), and 17 (10%) of these patients had elevated ferritin levels according to the MHF definition. In the control group with normal glucose tolerance, 11% also had hyperferritinemia based on the same ferritin cut-off. Multiple regression revealed a significant association between serum ferritin, male sex, 2-hour glucose value after the glucose load and waist circumference among the patients with IGT/T2DM, while there was no association with age or transferrin saturation.

**Conclusions:**

This study demonstrated a prevalence of metabolic hyperferritinemia in one out of ten people with IGT/T2DM; however, a comparable level of serum ferritin was also detected in the matched control group. Only one person in the IGT/T2DM group had ferritin levels higher than 550 mmol/L. Thus, hyperferritinemia is prevalent in this unselected Norwegian cohort. A relationship between s-ferritin and metabolic syndrome parameters was found in patients with IGT/T2DM, but hyperferritinemia is likely not related to iron overload.

## Background

In the last twenty-five years, an increasing number of publications have addressed hyperferritinemia in relation to metabolic syndrome, [1]– [3] and a recent consensus statement has proposed the term metabolic hyperferritinemia (MHF) to define this entity and standardize its nomenclature. [4] There has previously been debate regarding the presence of iron overload in patients with metabolic syndrome and hyperferritinemia. The new classification has an accompanying subclassification based on the levels of ferritin and the presence of hepatic iron storage and overt end-organ damage, as well as suggested diagnostic criteria. In short, serum ferritin is considered to reflect body iron stores and is suggested as the main marker to define the subclassification of MHF, particularly when MRI or liver biopsies are unavailable or not feasible. The clinical impact of hyperferritinemia is likely a result of oxidative stress, where excess iron generates free radicals through the Fenton process, which has in turn been linked to oxidized LDL, arterial plaque formation and atherosclerosis in cardiovascular disease, [5]and to end organ damage in the liver by iron mediated cell death, ferroptosis. [6].

Ferritin is also an acute phase protein, and the association between metabolic syndrome and hyperferritinemia is most apparent when correcting for ongoing inflammation. [7] A number of population-based studies have demonstrated an association between ferritin levels and insulin resistance; [8], [9] however, only a few studies have reported according to the new MHF classification. [10, 11] In two European cohorts the prevalence of hyperferritinemia was 12.5% and 13%, of which 81% in the last cohort were classified as MHF. [11, 12].

The Trøndelag Health Study (HUNT) [13, 14] are a series of population-based studies in central Norway. According to the HUNT1 Survey (1984-86), the prevalence of known diabetes mellitus (DM) among people older than 20 years was 2.6% in men and 3.2% in women. Eleven years later, the prevalence was 3.2% in both sexes and the prevalence of obesity (BMI *≥* 30) doubled among men during this period. [15].

In light of the new definition of MHF, it is interesting to examine the prevalence of MHF and the relationship between serum ferritin and insulin resistance in a Norwegian population.

## Methods

### Aim

We wanted to investigate (1) the prevalence of metabolic hyperferritinemia among persons with impaired glucose tolerance or T2DM in this population and (2) whether serum ferritin is related to metabolic markers.

### Population

The second Nord-Trøndelag Health Survey (HUNT2) was conducted between 1995 and 1997 in Nord-Trøndelag County, Norway. [16] HUNT2 was a cross-sectional population-based study comprising extensive information on exposure and clinical status from 66.7% of men (*n* = 30,860) and 75.5% of women (*n* = 35,280) aged ≥ 20 years in the county.

The HUNT2 Survey was applied as a reference population in our study regarding BMI (mean values in nondiabetic women; 26.2 kg/m^2^, nondiabetic men; 26.4 kg/m^2^, diabetic women 29.9 kg/m^2^ and diabetic men 28.0 kg/m^2^).

Verdal is a municipality in Nord-Trøndelag County, with a population of approximately 13,500 people. A total of 70.3% (7,136 persons) of the inhabitants in Verdal, aged 20 years and above, attended HUNT2 in 1996. In 2004, a total of 1,000 women and 1,000 men aged 20 years and older who were living in Verdal were randomly selected from the Public Registration Office and invited to participate in an oral glucose tolerance test (OGTT) according to the WHO 1999. [17] The study was approved by the Regional Ethics Board (REK) under application numbers 152/95/AH/JGE, 4.2006.250 and 881,426 under concession number 15/01521-11/GRA.

Out of the 2000 invited participants 1,115 completed the OGTT. A total of 176 patients were diagnosed with impaired glucose tolerance (IGT) or previously unknown or known T2DM and composed the study population. A control group comprising 100 age- and sex-matched individuals from the same region with a normal OGTT, fasting glucose < 5.6 and CRP < 5 was retrospectively created using data from the 2006 HUNT3 cohort. All participants provided written informed consent. Data from Statistics Norway provided information on the representativity of our study population compared to the general Norwegian population.

*Examination*: Height and weight were measured with the participants wearing light clothes without shoes, height to the nearest cm and weight to the nearest 0.5 kg. Waist circumference was measured horizontally at the height of the umbilicus to the nearest 1.0 cm with the participant standing and the arms hanging relaxed. *Laboratory tests*: The persons invited, except those with known diabetes (KDM) (according to the participants’ self-reported information on diagnosis and medication), attended after overnight fasting (for at least 8 h). They met between 07.00 am and 11.00 am for a fasting capillary whole blood glucose test measured on a HemoCue 201+^®^ Analyser (HemoCue AB, Ängelholm, Sweden). Daily calibration and control of HemoCue 201 + were performed according to the manufacturer’s protocol. Those with a fasting capillary whole blood glucose < 6.1 mmol/Lhad an OGTT according to WHO 1999, [17] with a 2-hour blood glucose measured by the same technique. Height, weight and waist circumference were measured in the same way as described for HUNT2. While the participants were resting for two hours between the fasting and two-hour blood glucose measurements, they completed a health-related questionnaire. At the end of the session, the participants were informed of their glycemic status according to the 1999 WHO criteria. [17] Persons with IGT were defined by a fasting glucose < 6.1 mmol/L and 2-hour post glucose values ≥ 7.8 mmol/L and < 11.1 mmol/L, while T2DM was either known prior to screening or detected at screening by fasting glucose ≥ 6.1 mmol/L or 2-hour post glucose ≥ 11.1 mmol/L or both, in the same manner as previously reported. [18].

**Fig. 1:**
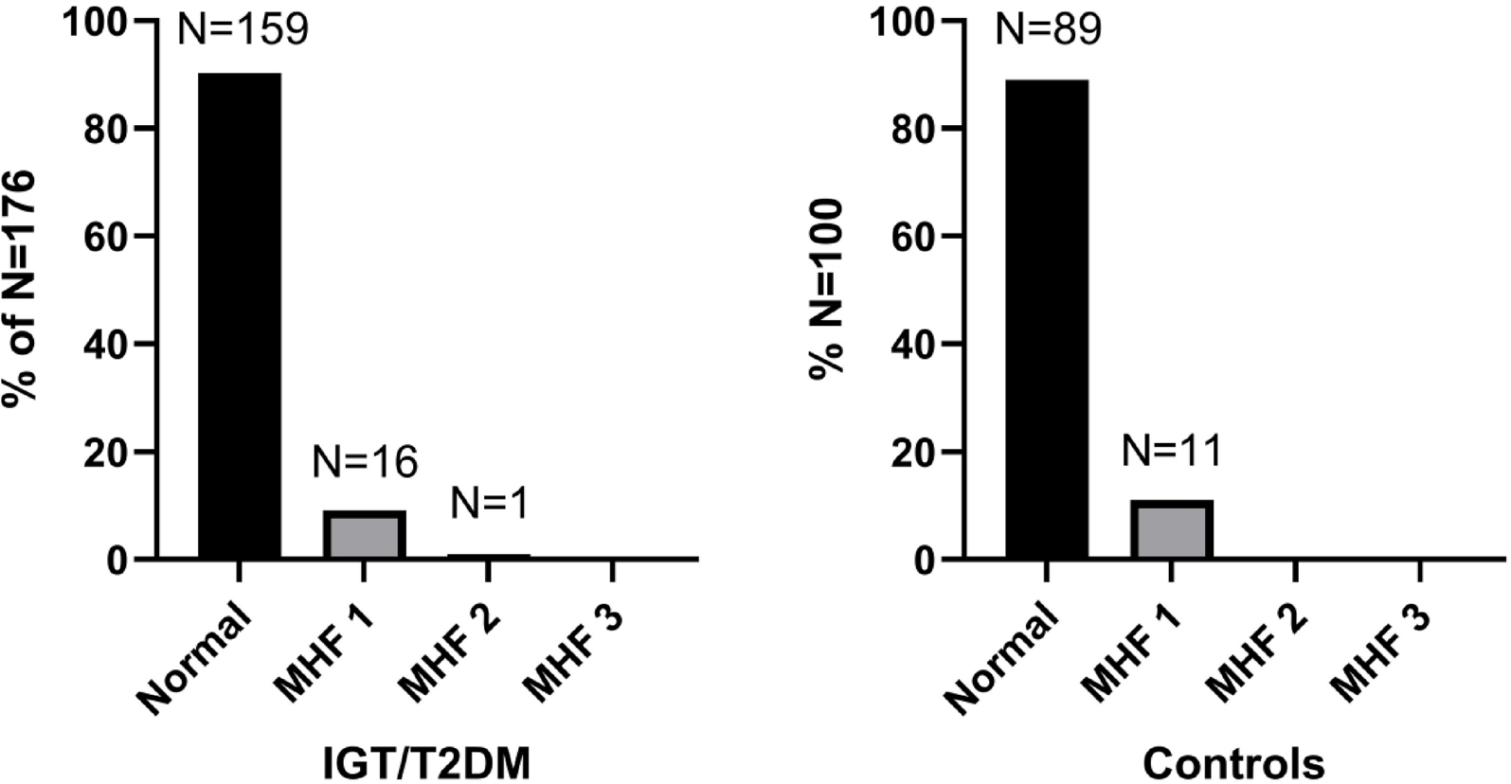
Distribution of normal iron metabolism and metabolic hyperferritinemia in patients with Impaired glucose tolerance or Type 2 Diabetes mellitus and healthy controls.

Individuals with IGT or T2DM were asked to return within a week for blood sampling for analysis of anti-GAD, C-peptide, HbA_1c_, ferritin, s-iron, TIBC and CRP. Tests were analysed at the Department of Clinical Chemistry at Levanger Hospital. C-reactive protein, s-iron and s-ferritin were measured by an Architect ci8200 (Abbott). Transferrin saturation was estimated by the equation s-iron/TIBC x 100. Cut-off values for elevated s-ferritin were 300 µg/l for men and 200 µg/l for women in order to adhere to the proposed MHF definition by Valenti. [4] HbA1c was reported as a percentage at the time of sampling and not according to the current standard of mmol/mol (Fig. 1).

.

### Statistical analysis

A normal distribution of the data was verified by normality plots. All normally distributed measurements are presented as the mean and standard deviation (SD). Normally distributed parameters were compared using Student’s t test. Markers found to be associated with hyperferrinemia and IGT/T2DM in a previous study by our group [19] and other markers known to be strongly linked to the metabolic syndrome collected in the HUNT trial were included in the univariate multiple linear regression. Variables included in the univariate analysis were serum ferritin, waist circumference, gender, 2-h OGTT, C-peptide, age, serum iron and transferrin saturation. Multiple linear regression analysis was used to investigate the associations between s-ferritin and other variables using the F-statistic in a stepwise method with a F to enter ≤ 0.05 and F to remove > 0.1. All tests were 2-sided, and a p value < 0.05 was considered to indicate statistical significance. No adjustments for multiple testing were performed. All analyses were performed with IBM SPSS 25 (IBM Corporation, Armonk, NY, USA).

## Results

Of the 1,115 persons attending the study, 176 fulfilled the criteria for IGT or T2DM; of these, 73 were male, and 103 were female. The mean age was 59 years. The clinical and biochemical characteristics of the patients are shown in Table 1.

**Table 1:** Characteristics of the study population

		All IGT/T2DM *N*=176	Male *N*=73	Female *N*=103	Reference values
		Mean (SD)	Mean (SD)	Mean (SD)	
Age	*N*=176	**59.2** (14.5)	62.3 (13.7)	57.0 (14.6)	
Waist circumference	*N*=171	**97.3** (11.4)	105 (10.6)	95.1 (11.4)	Male: <94 cm Female: <80 cm
C-peptide	*N*=167	**908** (389)	918 (396)	900 (387)	240–720 pmol/L
Fasting glucose	*N*=176	**5.9** (1.4)	6.2 (1.8)	5.6 (1.0)	3.6–5.8.6.8 mmol/L
2-hour glucose (OGTT)	*N*=118	**9.2** (1.2)	9.2 (1.0)	9.2 (1.3)	< 7.8 mmol/L
HbA_1C_	*N*=172	**6.0** (0.9)	6.3 (1.1)	5.8 (0.7)	3.5–6.5.5.5%
Fasting iron	*N*=172	**17** (5.4)	16.7 (5.1)	17.3 (5.6)	9–34 μmol/L
Transferrin saturation	*N*=171	**28** (9.5)	29 (8.8)	28 (10.1)	15–45%
S-Ferritin	*N*=176	**124** (97)	169 (110)	92 (72)	Male: <300 μg/L Female: <200μg/L
		**All controls** *N*=100	**Male** *N*=40	**Female** *N*=60	**Referance values**
Age	*N*=100	**57**,**1** (14)	58,6 (14)	56,1 (14)	
S-Ferritin	*N*=100	**119** (99)	149 (89)	99.1 (100)	Male: <300μg/L Female: <200 μg/L

The study population expressed markers typical of insulin resistance/T2DM. Waist circumference and BMI were elevated. 10% of the study population had MHF according to ferritin-based categories as proposed by Valenti et al. [4] with levels above 300 µg/L for males and 200 µg/L for females for a total of 17/176 (9/73 men and 8/103 women). The mean ferritin was 124 µg/L (SD 97) for the group as a whole and 169 µg/L (SD 110) and 92 µg/L (SD 72) for males and females, respectively. Sixteen of these patients could be classified as MHF grade I, one as grade II, and none as Grade III. Similarly, the number of persons with hyperferritinemia in the control group was 11/100 (3/40 males and 8/60 females), and all 11 would be MHF grade I based on ferritin cut-offs. The mean s-ferritin concentrations in the control group were 119 µg/L (SD 99), 149 µg/L (SD 89) and 99 µg/L (SD 100) for the whole group, men and women, respectively. There was no significant difference in the serum ferritin level between the patients with increased insulin resistance/T2DM and those in the control group (*N* = 276, mean 124 and 119 respectively, df = 274, *p* = 0.699). The fasting serum iron and transferrin saturation levels for the study population were within the reference limits.

Univariate linear regression analysis was performed for the study population and revealed significant associations between serum ferritin and male sex (F(1,174) = 30.881, *p* < 0.001, R^2^ = 0.151), waist circumference (F(1,169) = 20.596, *p* < 0.001, R^2^ = 0.109) and C-peptide (F(1,165) = 8.752, *p* = 0.004, R^2^ = 0.050) but not between serum ferritin and 2-hour fasting glucose (F(1,116) = 3.645, *p* = 0.059, R^2^ = 0.030), transferrin saturation (F(1,169) = 3.656, *p* = 0.058, R^2^ = 0.021), s-iron (F(1,170) = 2.349, *p* = 0.127, R^2^ = 0.014) or age (F(1,174) = 2.108, *p* = 0.148, R^2^ = 0.012).

**Fig. 2:**
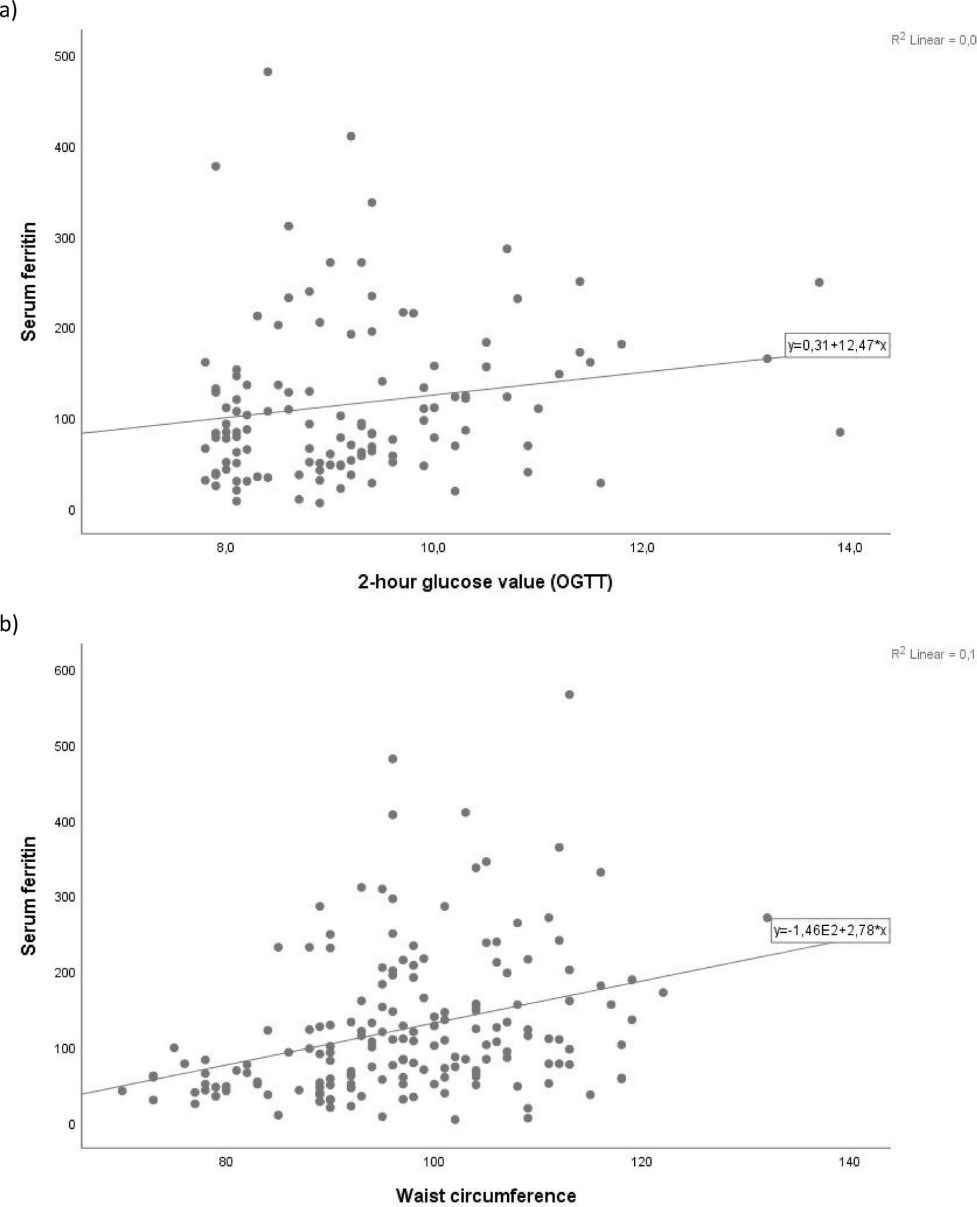
Univariate linear regression between ferritin and 2-hour Oral glucose tolerance test (**a**) and waist circumference (**b**)

Multiple linear regression analyses revealed that the significant predictors for serum ferritin according to the stepwise method (F(3,106) = 8.743, *p* < 0.001, R^2^ = 0.198) were male sex (*p* = 0.001), 2-hour glucose value (OGTT) (*p* = 0.030) and waist circumference (*p* = 0.045). Linear regression for the two last variables are presented in Fig. 2. Thus, serum iron and transferrin saturation had no predictive value for serum ferritin in this population.

## Discussion

Increased serum ferritin has been associated with a variety of conditions typical of metabolic syndrome. [19–21] A recent consensus paper by Valenti et al. summarized the current knowledge in the field and suggested a novel definition of this entity. [4].

We have previously published data showing an association between hyperferritinemia without overt iron overload and insulin resistance. [19] In this prior study on patients referred to a hospital outpatient clinic, ferritin levels were slightly elevated; however, the study population was small and consisted mainly of men. In the present study, we evaluated data from a population-based survey from a broader perspective. Impaired glucose regulation/type 2 diabetes (IGT or T2DM) were used to select a population with increased insulin resistance. The prevalence of MHF was 10% in this population. Surprisingly, a similar degree of hyperferritinemia was found for the matched control population without impaired glucose tolerance. Multiple regression analysis revealed sex, 2-hour glucose value and waist circumference as significant predictors of serum ferritin, while fasting serum iron and transferrin saturation had no such predictive value. In the univariate linear regression C-peptide also showed a correlation with ferrintin and would be a simpler alternative for large scale studies compared to OGTT. Males had higher ferritin values compared to females, a phenomenon usually explained by women’s bleeding periods. According to the IDF criteria for metabolic syndrome [22], there was a significant association between metabolic syndrome elements and serum ferritin in the study population, which is consistent with previously published literature. [7–9] There was no indication that our population had iron overload based on serum transferrin saturation and fasting serum iron levels. This is in line with the novel definition of MHF, where MHF grade I is defined by ferritin up to 550 mmol/l and where iron overload is not expected. [4] Our control group was selected from the same population cohort as our study population; however, they were selected retrospectively based on a normal fasting glucose level, a normal glucose tolerance test and CRP < 5. Unfortunately, supplemental data on other markers of metabolic syndrome were not available for the control group. Thus, the high ferritin levels in the control group are unexplained but could be related to other factors of metabolic syndrome, like waist circumference, in the absence of IGT/DMII. The WHO has concluded that ferritin levels > 200 µg/L for men and > 150 µg/L for women are appropriate for defining the risk of iron overload in otherwise healthy populations and > 500 µg/L for unhealthy individuals (WHO 2020),[] while > 300 µg/L for men and > 200 µg/L for women are suggested as thresholds used in the definition of MHF. [4] The highest registered s-ferritin level in our population was 566 µg/L, which is just above the lower limit of MHF grade II. Notably, in the Scripps/Kaiser Hemochromatosis Study, a screening for ferritin above 1000 µg/L detected the majority of patients who would be clinically affected by iron overload, [24] and the same was suggested by Valenti et al., as this is set as the threshold for MHF grade III. [4].

This study further underlines the association between serum ferritin and insulin resistance or other parameters of metabolic syndrome, particularly for males. The prevalence of MHF is approximately 10% of the population with increased insulin resistance or type 2 diabetes. Typically, there is mild hyperferritinemia, usually with a serum ferritin concentration < 550 µg/L, and, rarely, overt iron overload. A recent study indicated that the population with MHF could have adverse outcomes compared to patients without hyperferritinemia both with and without metabolic dysfunction,[10] but this needs to be evaluated in prospective trials. Iron-driven oxidative stress is suggested to play a part in end organ damage in the cardiovascular system. [5] Also, iron mediated cell death, ferroptosis, is a suggested pathological mechanisms involved in progression of liver disease from steatosis to steatohepatitis, fibrosis and ultimately hepatocellular carcinoma. [6, 25] In line with this, increasing levels of ferritin are associated with a higher Liver risk score and advanced fibrosis. [12] However, efforts to reduce serum ferritin through venesection have not been successful and should be avoided. [26].

This study has several limitations, the greatest being the creation of a control group from a later HUNT cohort compared to the main group with IGT/T2DM. The control group also lacks structured information related to the metabolic syndrome, making interpretation difficult. The screening for diabetes mellitus and oral glucose tolerance test was performed using capillary whole blood which could lead to misclassification of subjects. A small number of patients have missing data regarding some of the variables as presented in Table 1. Also, the patients who had previously known T2DM did not perform the OGTT, resulting in missing data for parameter for a subgroup of patients. However, we choose to present the data as complete as possible rather than to exclude subjects with missing data.

In our study, we used waist circumference, OGTT results, and C-peptide levels as valid markers of insulin resistance and T2DM. We found that MHF levels were comparable to those presented for other European cohorts. [11, 12] Our findings support the usefulness and ferritin cut-off points of the novel definition of metabolic hyperferritinemia, and in particular, MHF grade I seems prevalent. In line with the increasing global prevalence of insulin resistance and metabolic syndrome, MHF should be further evaluated in future population-based studies and clinical trials.

## Data Availability

The Trøndelag Health Study (HUNT) has invited persons aged 13 - 100 years to four surveysbetween 1984 and 2019. Comprehensive data from more than 140,000 persons havingparticipated at least once and biological material from 78,000 persons are collected. The dataare stored in HUNT databank and biological material in HUNT biobank. HUNT ResearchCentre has permission from the Norwegian Data Inspectorate to store and handle these data.The key identification in the data base is the personal identification number given to allNorwegians at birth or immigration, whilst de-identified data are sent to researchers upon approval of a research protocol by the Regional Ethical Committee and HUNT Research Centre. To protect participants’ privacy, HUNT Research Centre aims to limit storage of dataoutside HUNT databank, and cannot deposit data in open repositories. HUNT databank hasprecise information on all data exported to different projects and are able to reproduce theseon request. There are no restrictions regarding data export given approval of applications to HUNT Research Centre. For more information see: http://www.ntnu.edu/hunt/data.
